# Supplementary material for: Folding correctors can restore CFTR posttranslational folding landscape by allosteric domain–domain coupling
Source: Nat Commun. 2023 Oct 27;14:6868. doi: 10.1038/s41467-023-42586-8 (PMC10611759; doi:10.1038/s41467-023-42586-8)
Supplement: Supplementary file 3 — Description of additional supplementary files [file 41467_2023_42586_MOESM3_ESM.pdf]

Description of additional supplementary files

File name: Supplementary Data 1

Description: Sequence information of sense and anti-sense oligonucleotides for CFTR, MRP1, ABCC6 and their NBD1s mutagenesis, as well as for CFTR QPCR.
