# Supplementary material for: Folding correctors can restore CFTR posttranslational folding landscape by allosteric domain–domain coupling
Source: Nat Commun. 2023 Oct 27;14:6868. doi: 10.1038/s41467-023-42586-8 (PMC10611759; doi:10.1038/s41467-023-42586-8)
Supplement: Supplementary file 5 — Reporting Summary [file 41467_2023_42586_MOESM5_ESM.pdf]

nature portfolio

Corresponding author(s): Naoto Soya, Gergely L. Lukacs

Last updated by author(s): Oct 5, 2023

Reporting Summary

Nature Portfolio wishes to improve the reproducibility of the work that we publish. This form provides structure for consistency and transparency in reporting. For further information on Nature Portfolio policies, see our Editorial Policies and the Editorial Policy Checklist.

Statistics

For all statistical analyses, confirm that the following items are present in the figure legend, table legend, main text, or Methods section.

n/a	Confirmed	
☐	☒	The exact sample size (*n*) for each experimental group/condition, given as a discrete number and unit of measurement
☐	☒	A statement on whether measurements were taken from distinct samples or whether the same sample was measured repeatedly
☐	☒	The statistical test(s) used AND whether they are one- or two-sided<br>*Only common tests should be described solely by name; describe more complex techniques in the Methods section.*
☒	☐	A description of all covariates tested
☐	☒	A description of any assumptions or corrections, such as tests of normality and adjustment for multiple comparisons
☐	☒	A full description of the statistical parameters including central tendency (e.g. means) or other basic estimates (e.g. regression coefficient) AND variation (e.g. standard deviation) or associated estimates of uncertainty (e.g. confidence intervals)
☐	☒	For null hypothesis testing, the test statistic (e.g. *F*, *t*, *r*) with confidence intervals, effect sizes, degrees of freedom and *P* value noted<br>*Give P values as exact values whenever suitable.*
☒	☐	For Bayesian analysis, information on the choice of priors and Markov chain Monte Carlo settings
☒	☐	For hierarchical and complex designs, identification of the appropriate level for tests and full reporting of outcomes
☒	☐	Estimates of effect sizes (e.g. Cohen's *d*, Pearson's *r*), indicating how they were calculated

Our web collection on statistics for biologists contains articles on many of the points above.

Software and code

Policy information about availability of computer code

Data collection

1. Image Lab (Immunoblotting)
2. Xcalibur 2.1 and 4.3 (Orbitrap MS)

Data analysis

1. Excel and Graphpad Prism 9.4.1 (graphs and statistical analysis)
2. OriginPro 8 (Dose-response fitting)
3. ImageJ 1.50i (Immunoblotting analysis)
4. Pymol 2.5.4 (Structural analysis of HDX-MS results and MD analysis)
5. Modeller 9.23 (homology modelling)
6. VMD (MD analysis)
7. ClustalW (sequence alignments)
8. HDExaminer 2.5 and 3.3 (HDX-MS analysis)
9. HX-Express 2 (HDX-MS bimodal peak deconvolution, available at https://www.hxms.com/HXExpress/)
10. Proteome Discoverer 2.3 and 2.4 SP1 (MS/MS data analysis for peptide identification)
11. MDAnalysis (MD analysis)

For manuscripts utilizing custom algorithms or software that are central to the research but not yet described in published literature, software must be made available to editors and reviewers. We strongly encourage code deposition in a community repository (e.g. GitHub). See the Nature Portfolio guidelines for submitting code & software for further information.

Data

Policy information about availability of data

All manuscripts must include a data availability statement. This statement should provide the following information, where applicable:

- Accession codes, unique identifiers, or web links for publicly available datasets
- A description of any restrictions on data availability
- For clinical datasets or third party data, please ensure that the statement adheres to our policy

All data presented in this study are available within the figures and in the Supplementary Information, including the Supplementary Data 1 and Source Data files linked to this article. The source data underlying Fig. 1b-d, Fig. 2b-j, Fig. 3c-h, Fig. 4a-e, Fig. 5b-i, Fig.6a-c, and Supplementary Figures: Fig. 1b-h, Fig.2a-d, Fig.3a-d, f, Fig. 4a-b, Fig. 5a-b, Fig. 6c-d, Fig. 7a-d, Fig. 8a-c, Fig. 10c, Fig.11a-e, and Fig. 12a-c, ass well ass all the gel and auto-radiograpy images are included in the Source Data files. The HDX-MS raw data set have been deposited to the ProteomeXchange Consortium via the PRIDE partner repository (dataset identifier PXD042481). Files for homology modeling and MD simulations are available at zenodo https://zenodo.org/record/8388593. Other data are available from the corresponding author upon reasonable request.

Research involving human participants, their data, or biological material

Policy information about studies with human participants or human data. See also policy information about sex, gender (identity/presentation), and sexual orientation and race, ethnicity and racism.

Reporting on sex and gender	n/a
Reporting on race, ethnicity, or other socially relevant groupings	n/a
Population characteristics	n/a
Recruitment	n/a
Ethics oversight	n/a

Note that full information on the approval of the study protocol must also be provided in the manuscript.

Field-specific reporting

Please select the one below that is the best fit for your research. If you are not sure, read the appropriate sections before making your selection.

☒ Life sciences ☐ Behavioural & social sciences ☐ Ecological, evolutionary & environmental sciences

For a reference copy of the document with all sections, see nature.com/documents/nr-reporting-summary-flat.pdf

Life sciences study design

All studies must disclose on these points even when the disclosure is negative.

Sample size	Sample size was chosen on the basis of previous studies (e.g. Nature Chem Biol and Nature Med
Data exclusions	There are no data point exclusions. Occasionally entire experiment was excluded in case the cell viability was compromised (<5% of all experiments.)
Replication	All experiments were performed at least three times to calculate SEM or SD in the data analysis.
Randomization	This is not relevant to the study. Purified CFTR- variants or their NBD1 were exposed to HDX-MS experiments. In cell based assay the ABCC-transporters expression, folding efficiency, stability and drug sensitivity were measured.
Blinding	Blinding was not necessary, as none of the experiments have soft endpoints.

Reporting for specific materials, systems and methods

We require information from authors about some types of materials, experimental systems and methods used in many studies. Here, indicate whether each material, system or method listed is relevant to your study. If you are not sure if a list item applies to your research, read the appropriate section before selecting a response.

Materials & experimental systems

n/a	Involved in the study	
☐	☒	Antibodies
☐	☒	Eukaryotic cell lines
☒	☐	Palaeontology and archaeology
☒	☐	Animals and other organisms
☒	☐	Clinical data
☒	☐	Dual use research of concern
☒	☐	Plants

Methods

n/a	Involved in the study	
☒	☐	ChIP-seq
☒	☐	Flow cytometry
☒	☐	MRI-based neuroimaging

Antibodies

Antibodies used

1. Monoclonal mouse ani-HA Ab from BioLegend (1:2000, #901515)
2. Anti-CFTR Ab L12B4 (#MAB3484, 1:1000, recognizing residues 386-412 of the NBD1) and M3A7 (#05-583, 1:500, recognizing residues 1365-1395 at the C terminus of the NBD2) from Millipore Bioscience Research Reagents (Temecula, CA) or provided by J. Riordan/M. Gentzsch laboratory (University of North Carolina, Chapel Hill, NC)
3. 660 Ab (1:2000, recognizes NBD1) provided by J. Riordan and CF Foundation via the CFTR Antibodies Distribution Program
4. Mouse monoclonal anti-CFTR Ab MM13-4 (#05-581, 1:500, specific to the N-terminal a.a. 25-36) f rom Millipore (Billerica, MA)
5. Anti-human MRP1Abs: QCRL-1 (#SC-18835, 1:1000, specific for a.a. 918-924) from Santa Cruz Biotechnology
6. MRPr1 (#Ab3368, 1:1000, specific for a.a. 238-247, Abcam), 897.2 (1:1000, specific for a.a. 1316-1388) and 643.4 (1:1000, specific for MRP1NBD2) provided by X.B. Chang, Mayo Clinic College of Medicine (Scottsdale, AZ)
7. Anti-ABCC6 Ab (1:1000, #D9D1F) from Cell Signaling Technology (Danvers, MA)
8. Anti-Na/K-ATPase Ab (1:5000, #SC-48345) from Santa Cruz Biotechnology
9. HRP-conjugated secondary Abs: sheep anti-mouse IgG (1:2000, GE Healthcare, NXA931)
10. Goat Anti-Rat IgG (1:2000, Jackson ImmunoResearch, 112-035-003)
11. F(ab')$_2$ Fragment Goat Anti-Mouse IgG (1:1000, Jackson ImmunoResearch, 115-036-003)

Validation

1. Manufacturer's website BioLegend: This second-generation anti-HA antibody is an excellent substitute for the 12CA5 monoclonal antibody. The HA.11 antibody recognizes the influenza hemagglutinin epitope (YPYDVPDYA) which has been used extensively as a general epitope tag in expression vectors. The extreme specificity of the antibody allows unambiguous identification and quantitative analysis of the tagged protein. The HA.11 antibody recognizes HA epitopes located in the middle of protein sequences as well as at the N- or C-terminus.
2. Manufacturer's website Millipore Bioscience:
M3A7 (MAB3480) Ab: Recognizes an epitope at the C-terminal end of the second nucleotide binding in the region of residues 1370 to 1380. Clone M3A7 detects level of CFTR & has been published & validated for use in IC, IP & WB. IP, IF, T84 adenocarcinoma cells are a good positive control. We also used CFTR-/- cells as negative control to test CFTR Abs specificity in general. L12B4 (MAB3484) Ab: Recognizes an epitope at the cytoplasmic region preceding the first nucleotide binding domain between residues 386 to 412. Recognizes CFTR, Mr 170kDa and two additional proteins at ~ Mr 100 and 140kDa. clone L12B4 is an antibody against Cystic Fibrosis Transmembrane Conductance Regulator for use in IC, IP & WB. Western blot: 1-10µg/mL, note do not boil the lysate, incubate at 80C for 30 minutes prior to running SDS-PAGE. CTFR aggregates upon boiling. Antibody recognizes CFTR at 170kDa and two additional proteins at 100 & 140kDa.
3. 660 Ab: IgG2b, recognizes an epitope of a.a.484-589 in the NBD1 of CFTR. Validation was performed on CFTR expressing and deficient cells. Provided by J. Riordan and CF Foundation via the CFTR Antibodies Distribution Program
4. Manufacturer's website Millipore Bioscience: MM13-4 (MAB3482): Synthetic peptide (RKGYRQRLELSD) corresponding to residues 25-36 of human cystic fibrosis transmembrane conductance regulator (CFTR). Detect Cystic Fibrosis Transmembrane Conductance Regulator using this Anti-Cystic Fibrosis Transmembrane Conductance Regulator Antibody, clone MM13-4 validated for use in IC, IP & WB. Western blot: 10 µg/mL, recognizes CFTR (150-170kDa) and two additional proteins at 52kDa and 38kDa.
5. Manufacturer's website Santa Cruz: MRP1 Antibody (QCRL-1) is an IgG1 κ mouse monoclonal MRP1 Antibody (QCRL-1) is recommended for detection of an epitope between amino acids 918-924 of MRP1 of human origin by WB, IP, IF, IHC(P) and FCM, MRP1 raised against a human small cell lung cancer cell line H69AR.
6. Manufacturer's website Abcam: Rat monoclonal [MRPr1] to MRP1 Specificity This antibody detects MRP 1. It does not cross-react with the human MDR 1 and MDR 3 P glycoprotein gene products. Tested applications Suitable for: WB, IHC-Fr, IHC-P, ICC/IF Species reactivity Reacts with: Human Immunogen Fusion protein corresponding to MRP1. Bacterial fusion protein of MRP containing a segment of 168 amino acids in the amino-proximal half of the protein.
7. Manufacturer's website Cell signaling Anti-ABCC6 (D9D1F) Rabbit mAb #10666: MRP6/ABCC6 (D9D1F) Rabbit mAb recognizes endogenous levels of total MRP6 protein. This antibody also cross-reacts with unidentified proteins of 65 kDa and 135 kDa. Monoclonal antibody is produced by immunizing animals with a synthetic peptide corresponding to residues near the carboxy terminus of human MRP6 protein. We also used non-transfected cells as control.
8. Manufacturer's website Santa Cruz Biotechnology: Anti-Na/K-ATPase ATP1A1/ATP1A2/ATP1A3 Antibody (H-3) is a mouse monoclonal IgG2b κ,against amino acids 551-850 mapping within an internal region of Na+/K+-ATPase α1 of human origin, ATP1A1/ATP1A2/ATP1A3 Antibody (H-3) is recommended for detection of Na+/K+-ATPase α1, 2 and 3 of mouse, rat and human origin by WB, IP, IF, IHC(P) and ELISA; also reactive with additional species, including canine
9. Manufacturer's website GE Healthcare, NXA931: HRP-conjugated secondary Abs: sheep anti-mouse: The antibodies are prepared by hyper-immunizing an animal with purified immunoglobulin fractions from normal human serum to produce high affinity antibodies. The pooled antiserum is used to produce an immunoglobulin preparation that is then affinity adsorbed to remove cross-reacting antibodies with the immunoglobulins of other species. These activities are thoroughly depleted to ensure species-specificity. Finally, to select for specific binding to human IgG, the antibodies are purified using an affinity column of human IgG. After washing to remove non-specific serum components and low affinity antibodies, the species-specific antibodies are eluted using carefully selected, mild conditions that minimize aggregation and preserve immunological activity, yet which will elute high affinity antibodies.

10. Manufacturer's website: Goat Anti-Rat IgG (1:2000, Jackson ImmunoResearch, 112-035-003): Based on immunoelectrophoresis and/or ELISA, the antibody reacts with whole molecule rat IgG. It also reacts with the light chains of other rat immunoglobulins. No antibody was detected against non-immunoglobulin serum proteins. The antibody may cross-react with immunoglobulins from other species. Whole IgG antibodies are isolated as intact molecules from antisera by immunoaffinity chromatography. They have an Fc portion and two antigen binding Fab portions joined together by disulfide bonds and therefore they are divalent. The average molecular weight is reported to be about 160 kDa.
11. Manufacturer's website: $F(ab')_2$ Fragment Goat Anti-Mouse IgG (1:1000, Jackson ImmunoResearch, 115-036-003) Based on immunoelectrophoresis and/or ELISA, the antibody reacts with whole molecule mouse IgG. It also reacts with the light chains of other mouse immunoglobulins. No antibody was detected against non-immunoglobulin serum proteins. The antibody may cross-react with immunoglobulins from other species. F(ab')2 fragment antibodies are generated by pepsin digestion of whole IgG antibodies to remove most of the Fc region while leaving some of the hinge region. F(ab')2 fragments have two antigen-binding Fab portions linked together by disulfide bonds and therefore they are divalent. The average molecular weight is about 110 kDa. They are used for specific applications, such as to avoid binding of secondary antibodies to live cells with Fc receptors or to Protein A or Protein G.
12. All secondary Abs specificity was tested in the presence of species relevant non-specific primary Ab.

Eukaryotic cell lines

Policy information about cell lines and Sex and Gender in Research

Cell line source(s)	The CFBE41o- cells were originally generated by and are a kind gift from Dr. Dieter Gruenert (University of California, San Francisco). BHK-21 cells were purchased from ATCC (#CCL-10).
Authentication	BHK-21 cells have been validated by ATCC and were obtained directly from ATCC. CFBE41o- were directly received from Dr. Dieter Gruenert and have not been authenticated in our lab. The CFBE41o- displays all ion transport properties characteristic of cystic fibrosis such as defective cAMP-dependent chloride transport and intact calcium-dependent chloride transport, and forms tight junctions to give a polarized epithelium as demonstrated previously in our lab (Veith et al Mol Biol Cell 2012 . 23(21):4188-202
Mycoplasma contamination	We routinely test cells for myocoplasma. All cells used in the study were tested negative.
Commonly misidentified lines (See ICLAC register)	No commonly misidentified cell lines were used.
